# Preparation, Thermal Properties and Decomposition Course of Highly Resistant Potato Starch Graft Poly(Cinnamyl Methacrylate) Materials

**DOI:** 10.3390/molecules30020376

**Published:** 2025-01-17

**Authors:** Marta Worzakowska

**Affiliations:** Department of Polymer Chemistry, Institute of Chemical Sciences, Faculty of Chemistry, Maria Curie-Skłodowska University, Gliniana 33 Street, 20-614 Lublin, Poland; marta.worzakowska@mail.umcs.pl; Tel.: +48-81-524-22-51

**Keywords:** potato starch, cinnamyl methacrylate, graft copolymers

## Abstract

The properties of starch graft poly(cinnamyl methacrylate) copolymers were presented. The “grafting from” method and different ratios of starch to methacrylic monomer were used. The copolymers with the maximum grafting percent (G: 55.3% ± 0.4) using a ratio of starch to methacrylic monomer of 1:3 were obtained. The heterogeneous, non-porous structure materials were prepared. They were characterized by significant lower swelling in polar solvents and moisture absorption but higher swelling in non-polar solvents compared to unmodified potato starch. The chemical resistance in acidic, alkaline and neutral environments for all the tested copolymers was significantly higher compared to the chemical resistance of potato starch. The tested copolymers decomposed in at least three main stages in inert conditions and in at least four main stages in oxidative conditions. Their pyrolysis with the emission of the mixture of volatiles such as aldehyde, acid, ester, alcohol, aromatic, alkene, alkane, H_2_O, CO_2_ and CO based on the TG/FTIR studies was proved. The oxidative decomposition included pyrolysis processes combined with oxidation and combustion reactions of volatiles and the formed residues. As a result, the emission of the unsaturated and saturated compounds with carbonyl, hydroxyl, carboxyl and/or ester groups, alkane, alkene, aromatics and its oxidized forms, H_2_O, CO_2_ and CO, was observed.

## 1. Introduction

Polysaccharide graft copolymers are valuable materials for many practical applications. They are widely applied as superabsorbents, flocculants, papermaking additives, oils recuperating agents, heavy metal ion removal, for sizing cotton, as mulch films and biodegradable polymers and also in the manufacture of molded plastics, ion-exchange resins, cosmetics, etc. [1,2,3,4,5]. Such great interest and practical application of this group of materials result from their properties, which can be modified in various ways. By modifying the polysaccharide in the graft polymerization process, it is possible to obtain more hydrophilic, more hydrophobic or amphiphilic materials compared to the initial polysaccharide. The additional advantages are the biodegradability, harmlessness, availability and the low price of polysaccharides. This makes them attractive materials for synthesis of new, more environmentally and human-friendly polymer materials compared to completely synthetic polymers [6,7,8,9,10].

Recently starch is a polysaccharide widely studied as a material suitable for the graft polymerization process. This natural carbohydrate polymer is a reserve substance found in plants and a source of energy for humans. It is widespread and can be obtained from various plant species, such as potatoes, rice, soybeans, corn, wheat, tapioca, etc. Chemically, starch is a macromolecular compound composed of two polymers, namely linear amylose and the branched amylopectin. The basic structural unit of starch is an α-D-glucopyranosyl unit. Each basic unit of amylose contains three hydroxyl groups in the structure, and amylopectin has two or three. A large number of hydroxyl groups in the macromolecule chains gives the polymer highly hydrophilic properties [8,9,10]. Due to the chemical structure of starch, it can be successfully modified in graft processes. This allows for obtaining materials with a wide range of properties in dependence on the used unsaturated monomer. Currently, the examples of the synthesis of starch graft copolymers using both hydrophilic and hydrophobic type compounds as the unsaturated monomers are known. Examples include the use of meth(acrylic) acids [11,12,13,14,15,16,17], acrylamide [18,19,20,21], itaconic acid [19,21], *N*-vinylformamide [22,23], maleic anhydride-diethylenetriamine [24], 2-hydroxyethyl methacrylate [25], 2-acrylamido-2-methylpropane sulfonic acid [25,26], acrylonitrile, etc., as hydrophilic-type unsaturated monomers. In turn, as hydrophobic-type unsaturated monomers styrene, vinyl acetate, methyl meth(acrylate)s, ethyl meth(acrylate)s, butyl meth(acrylate)s, hexyl meth(acrylate)s, benzyl meth(acrylate)s, cyclohexyl meth(acrylate)s, etc., are commonly applied [27,28,29,30,31,32,33,34,35,36]. We have recently undertaken the studies on the use of meth(acrylic) monomers obtained from terpene linear alcohols of natural origin for the starch graft modification process. We have obtained a series of starch graft copolymers with unique properties. These materials can be used practically as materials for applications where high chemical resistance and high resistance to solvents are required [37,38,39,40,41]. For this reason, we decided to continue our research.

This paper deals with the preparation and properties of starch graft copolymers obtained with the use of cinnamyl methacrylate monomer. This monomer in the acylation process of cinnamyl alcohol and methacryloyl chloride was synthesized. Cinnamyl alcohol is a α,β-unsaturated alcohol, naturally and abundantly occurring in essential oils like cinnamon and cassia. This alcohol can react readily with carboxylic acids, their derivatives or other esters to form ester compounds. Such ester compounds have many practical applications as additives to various food, cosmetic, pharmaceutical or medical products, fragrance compounds, as non-toxic plasticizers, monomers or enzyme inhibitors. Moreover, the presence of an ethylenic bond and an aromatic ring in its structure make it an interesting compound to research. Due to these, cinnamyl alcohol to the preparation of methacrylic ester and then the starch graft process is used. The effect of the grafting percent (G) on the solubility, chemical resistance, moisture absorption, thermal properties and the type of emitted volatiles under the decomposition process of the prepared starch graft copolymers in inert and oxidizing atmospheres was studied and discussed.

## 2. Results and Discussion

### 2.1. The Structures of Monomer and Obtained Starch Graft Copolymers

**Figure 1:**
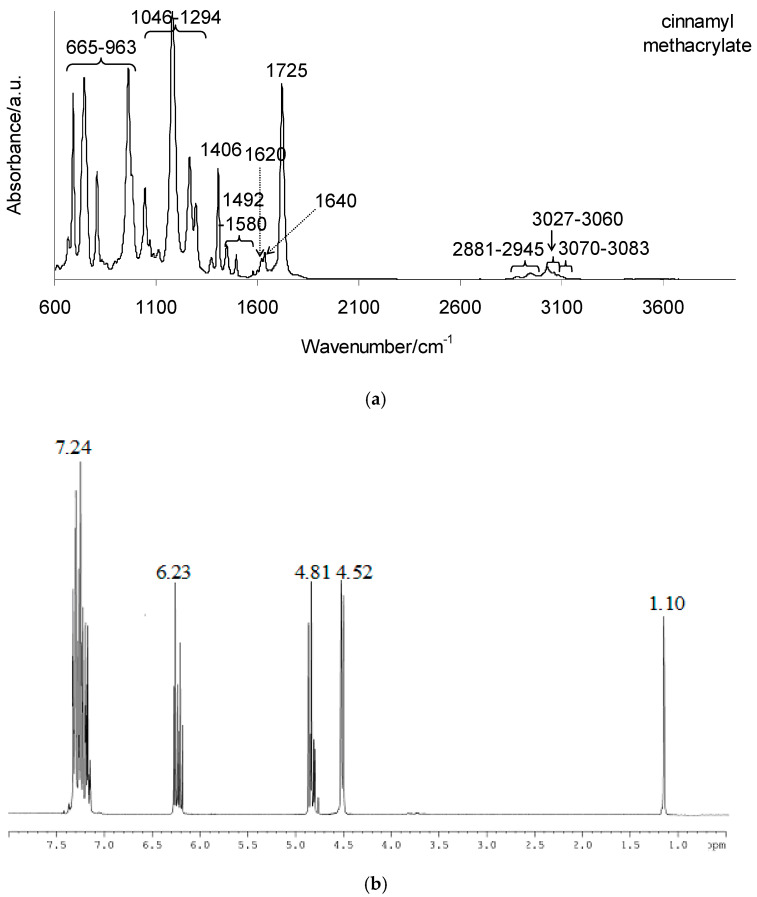
The FTIR (**a**) and ^1^HNMR (**b**) spectra for cinnamyl methacrylate.

The structure of the cinnamyl methacrylate monomer based on the spectroscopic methods was proved. The received results from the FTIR and ^1^H NMR for methacrylic monomer are presented in Figure 1. From these spectra, the following signals were assigned: FTIR (thin film, cm^−^^1^): 3083–3070 (ν C_Ar-H_), 3060–3027 (ν =C-H), 2945–2881 (ν C-H), 1725 (ν C=O), 1620 (ν C=C), 1640 (ν C=C), 1580–1492 (ν C=C_Ar_), 1406–1370 (δ C-H), 1294–1046 (ν C-O), 963–665 (γ =C-H). ^1^H NMR (300 MHz, CDCl_3_, δ ppm): 1.10 (s, -CH_3_) 4.52 (m, O-CH_2_-C), 4.81 (m, =CH_2_), 6.23 (m, =CH), 7.24 (m, -CH_Ar_).

**Figure 2:**
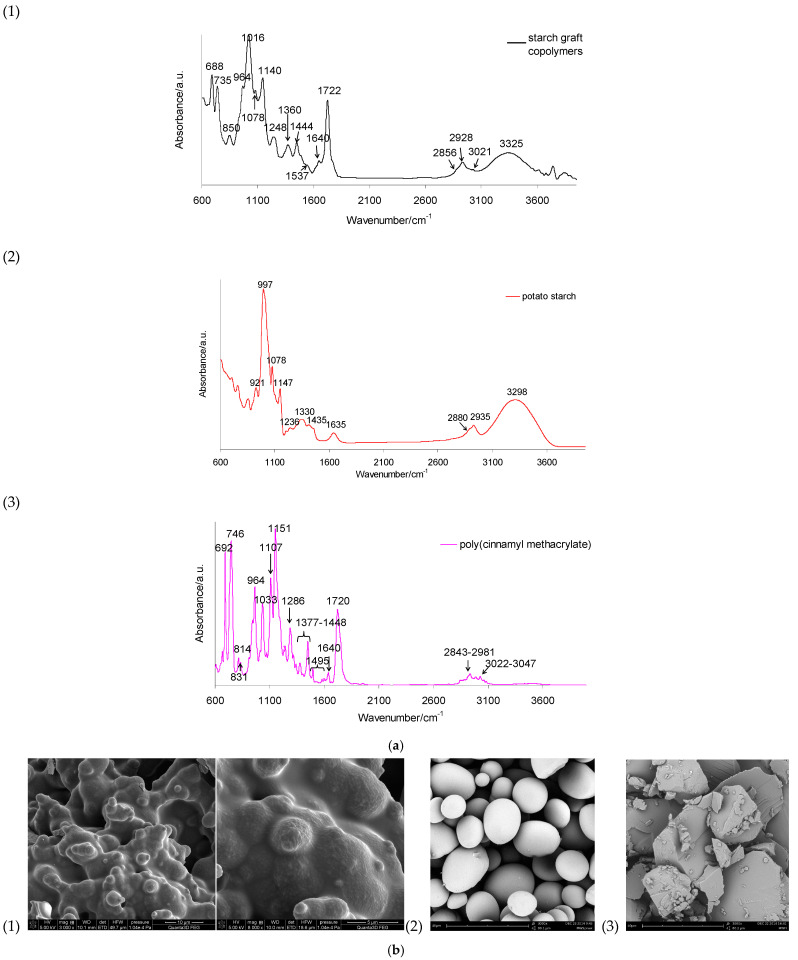

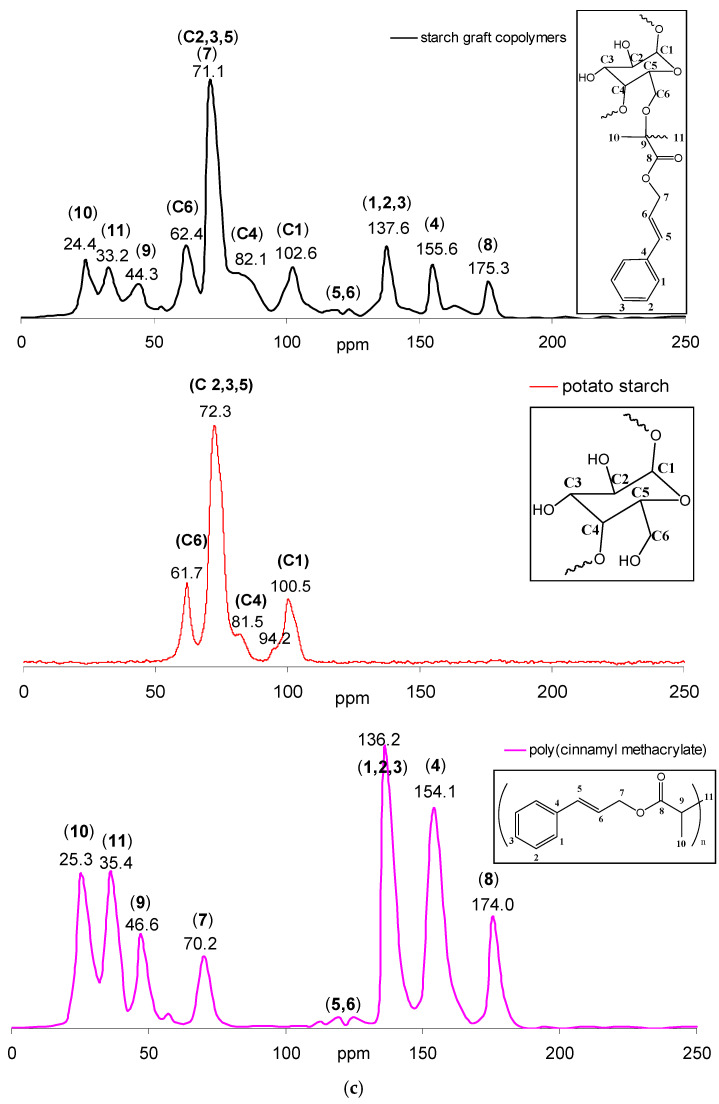
The FTIR spectra (**a**), SEM pictures (**b**) and ^13^C CP-MAS NMR (**c**) for the graft starch copolymers (1), potato starch (2) and poly(cinnamyl methacrylate) (3).

The structure of starch graft copolymers by the FTIR and ^13^C CP-MAS NMR was confirmed. The exemplary FTIR spectrum for starch graft copolymers is presented in Figure 2a. In addition, the FTIR spectra for unmodified potato starch (Figure 2a) and a homopolymer—poly(cinnamyl methacrylate)— are shown in Figure 2a in order to compare the results. As is clearly visible, the differences between these presented FTIR spectra are observed. The FTIR spectrum of unmodified potato starch shows the characteristic absorption bands at 3000–3600 cm^−^^1^ with a maximum at 3298 cm^−^^1^ which are related to the stretching vibrations of the OH groups in glucose units. The other absorption bands characteristic of starch at 2880–2935 cm^−^^1^ (the stretching vibrations of the C-H), at 1635 cm^−^^1^ (the OH bending of absorbed water), at 1330–1435 cm^−^^1^ (the deformation vibrations of the C-H) and at 921–1236 cm^−^^1^ (the stretching vibrations of the C-O-H and the C-O-C) are observed, Figure 2a. On the other hand, the FTIR spectrum of poly(cinnamyl methacrylate) presents all the characteristic absorption bands for the stretching vibrations of the C_Ar –H_ at 3022–3047 cm^−^^1^, the stretching vibrations of the C-H at 2843–2981 cm^−^^1^, the stretching vibrations of the C=O at 1720 cm^−^^1^, the stretching vibrations of the C=C (ethylenic bonds) at 1640 cm^−^^1^, the stretching vibrations of the C=C_Ar_ at 1495–1598 cm^−^^1^, the deformation vibrations of the C-H at 1377–1448 cm^−^^1^, the stretching vibrations of the C-O at 964–1286 cm^−^^1^ and the out-of-plane deformation vibrations of the C_Ar_-H and the =C-H at 692–831 cm^−^^1^. Furthermore, only a residual absorption band (of a very low intensity) associated with the stretching vibrations of the methacrylic double double bond at 1620 cm^−^^1^ is visible. This proves that methacrylic C=C bonds are more reactive in the applied experimental conditions compared to the reactivity of the ethylenic double double bonds (the absorption band at 1640 cm^−^^1^). Meanwhile, if we compare the above FTIR spectra with the FTIR spectrum for starch graft copolymers, the significant differences are noticed.

The FTIR spectra of the copolymers show the presence of the following absorption bands: the stretching vibrations of the OH (above 3100 cm^−^^1^ with the maximum at 3325 cm^−^^1^), the stretching vibrations of the C-H (2920–2856 cm^−^^1^), the stretching vibrations of the C=O (1722 cm^−^^1^), the stretching vibrations of the C=C (1640 cm^−^^1^), the stretching vibrations of the C=C_Ar_ at 1500–1537 cm^−^^1^, the deformation vibrations of the C-H (1444–1360 cm^−^^1^), the stretching vibrations of the C-O (1248–964 cm^−^^1^) and the out-of-plane deformation vibration of the =C-H and the C_Ar_-H (850–688 cm^−^^1^). A significant difference between FTIR spectra of starch and the prepared copolymers is the shift in the absorption band for the C-O-H groups in starch from 997 to 1016 cm^−^^1^ in copolymers. Also, the reduction in the intensity of the absorption bands responsible for the stretching vibrations of the OH groups in starch (above 3100 cm^−^^1^) is clearly observed. Moreover, the absence or shift in the absorption bands characteristic for the C-O groups in the homopolymer (region 964–1286 cm^−^^1^) towards lower wavenumber values as compared to these bands in the copolymer spectrum is confirmed, Figure 2a. This proves the successful incorporation of cinnamyl methacrylate chains through covalent bonds into the starch structure. In addition, the FTIR spectra of starch graft copolymers show the presence of only the absorption bands originating from ethylenic double bonds at 1640 cm^−^^1^. This confirms that cinnamyl methacrylate is attached to the starch backbone through more reactive methacrylic bonds.

The SEM pictures confirm that the morphology of the prepared copolymers is significantly different from the morphology of unmodified potato starch and poly(cinnamyl methacrylate), Figure 2b. The unmodified potato starch occurs in the form of egg-shaped balls with a smooth surface while the homopolymer poly(cinnamyl methacrylate) forms the blocks with a compact structure and a smooth surface without visible cavities. However, the morphology of the obtained starch graft copolymers is completely different. They have heterogeneous surfaces with different diameter craters. In addition, the surface of these copolymers look like growths without visible pores, shown in Figure 2b.

In turn, the ^13^C CP-MAS NMR spectra of starch graft copolymers, potato starch and poly(cinnamyl methacrylate) are presented in Figure 2c. Comparing these spectra, some differences between them are also visible, as is marked in Figure 2c.

**Scheme 1:**
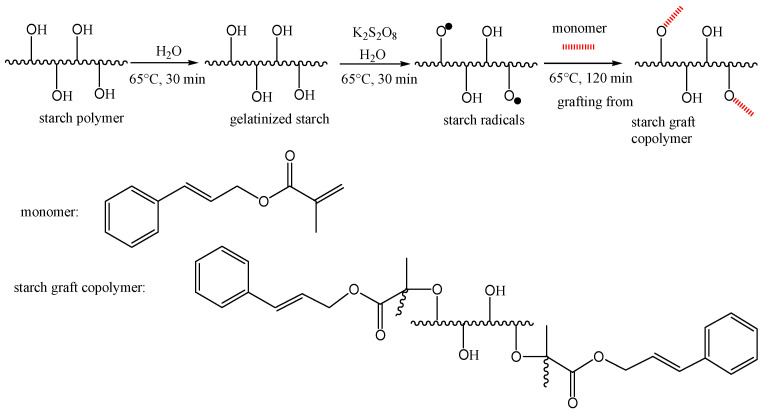
A simplified scheme of the preparation of starch graft copolymers.

Table 1 shows the values of the grafting percent (G,%). As it is well seen, the applied experimental grafting conditions allow for obtaining the copolymers with a different grafting percent. The G values depend on the used ratio of potato starch to cinnamyl methacrylate monomer. The maximum grafting percent (G,%) with the use of starch to monomer in the ratio of 1:3 is obtained, shown in Table 1. A simplified scheme of the preparation of starch graft copolymers is given in Scheme 1.

### 2.2. The Swelling and Moisture Absorption Properties

The prepared novel materials were tested to learn their behavior in the different polarity solvents. The obtained results are placed in Table 2. As is well visible, the swelling of the copolymers in polar type solvents depends on the G value and the solvent type. Generally, the swelling of the copolymers is definitely lower in polar solvents compared to the swelling of unmodified potato starch. Moreover, as the polarity of the solvents decreases, the swelling also decreases. In addition, as the G values grow, the swelling in polar solvents is reduced. This behavior of the obtained copolymers is predictable because the chemical modification of potato starch by cinnamyl methacrylate monomer causes the preparation of more hydrophobic type materials than unmodified potato starch. However, what is interesting is that the copolymers with low G values (copolymers 1 and 2) exhibit from ca. 3.2 times to ca. 5.0 times less swelling in polar solvents than unmodified potato starch. It suggests that the swelling properties of the copolymers are significantly modified by the incorporation of minor amounts of hydrophobic poly(cinnamyl methacrylate) chains into the potato starch structure. Moreover, the copolymers with higher G values (copolymers 3–5) by a definitely low swelling in polar solvents are characterized. Their swelling is ca. 5.5–7.8 times smaller than the swelling of potato starch. The swelling of the copolymers in non-polar solvents grows as the G values increase. Their swelling is from ca. 2.4 to ca. 4.4 times higher in toluene, from ca. 1.8 to ca. 3.1 higher in CCl_4_ and from ca. 1.0 to ca. 2.7 times higher in hexane compared to the swelling of unmodified potato starch. It is due to the presence of the poly(cinnamyl methacrylate) chains linked to the starch macromolecule. The presence of these chains blocks some of the hydrophilic groups of starch. Thus, the solvation of the obtained copolymers by non-polar solvents is better than the solvation of potato starch.

The very interesting results from the studies on the moisture absorption were obtained, as shown in Table 2. The moisture absorption for the copolymers with higher G values is significantly restricted. The copolymers 4 and 5 show ca. 10 times lower moisture absorption than unmodified potato starch. This leads to the conclusion that the chemical modification of potato starch by poly(cinnamyl methacrylate) allows for obtaining the materials with high resistance to moisture even when not all of the hydroxyl groups of starch by hydrophobic type chains are replaced. This situation is probably connected with the structure of poly(cinnamyl methacrylate) which contains “large” substituents such as benzene rings. This substituent tightly covers the surface of starch containing the OH groups. Also, it may be caused by the surface morphology of the copolymers. As confirmed by the SEM, the surface of the obtained copolymers is solidified with different diameters craters, shown in Figure 2b. The solvents can penetrate these craters and solvate the surface of the copolymers. However, due to the lack of porous and quite stable and compact structures, the swelling of the copolymers and moisture absorption are significantly limited.

### 2.3. The Chemical Stability

The chemical stability of the copolymers considerably changes after the chemical modification, shown in Table 3. All the studied copolymers exhibit higher chemical resistance in acidic, neutral and basic environments compared to the unmodified potato starch. As the G values grow, the chemical stability of the novel materials also grows. In addition, the chemical stability of the received materials is from ca. 3.7 times to ca. 12 times higher in acidic conditions, from ca. 4.5 times to ca. 13 times higher in neutral conditions and from ca. 1.4 times to ca. 1.9 times higher in basic conditions than unmodified potato starch. These results suggest that the incorporation of poly(cinnamyl methacrylate) chains into the structure of potato starch leads to the preparation of more resistant materials to hydrolysis, especially in acidic and neutral environments than potato starch. It makes them attractive materials for the manufacturing of the excipients for some drug delivery systems or drug coatings. Such materials will be chemically stable in an acidic environment found in the stomach. On the other hand, they will be partially decomposed in the alkaline environment in the duodenum, slowly releasing the drug. These copolymers can also be attractive materials used in papermaking like as a surface sizing agent or for coating the surface of paper to protect it against external factors.

### 2.4. The DSC Results

**Figure 3:**
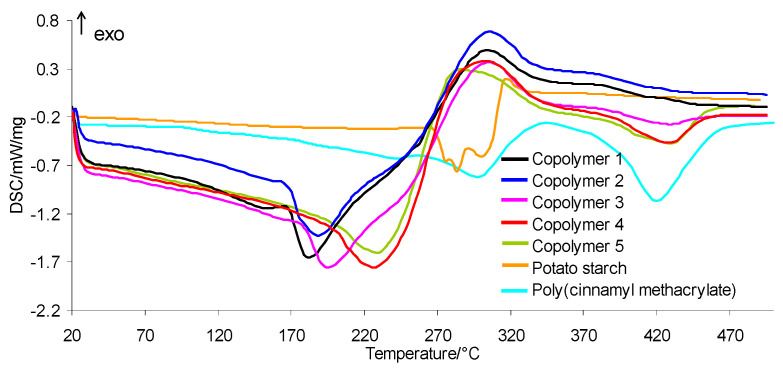
The DSC curves for starch graft copolymers, potato starch and poly(cinnamyl methacrylate).

The DSC curves of the prepared copolymers, potato starch and homopolymer are presented in Figure 3. As is well visible, three main DSC temperature ranges are observed. The first one, the endothermic DSC peak, starts from ca. 140–150 °C. Its maximum depends on the G values. As the G value increases, the maximum temperature of this DSC peak also increases. The maximum temperature is 182 °C for copolymer 1 and 230 °C for copolymer 5. Taking into account the structure of the tested materials and the observed dependencies, it is expected that this first, endothermic DSC peak is related to the decomposition of poly(cinnamyl methacrylate) chains from the copolymers. In addition, as the G value increases, the higher onset decomposition temperature of poly(cinnamyl methacrylate) from the tested copolymers is noticed. Moreover, below the temperature of 140–150 °C for copolymers with lower G values, a DSC peak of low intensity can be observed (copolymers 1–3). Its presence may indicate the decomposition of some component of the copolymer or evaporation of absorbed or bound water by the material. However, above the temperature of 270 °C, this endothermic DSC peak directly transforms into an exothermic signal with a maximum temperature from 285 °C to 307 °C. In this temperature range, as can be seen from Figure 3, both unmodified potato starch and the homopolymer poly(cinnamyl methacrylate) decompose under the influence of temperature. Their decomposition as an endothermic DSC peak, with two maxima at 283 °C and at 301 °C for unmodified potato starch, and at 300 °C for the poly(cinnamyl methacrylate) homopolymer, is described. In the case of starch graft copolymers, the presence of an exothermic DSC signal may indicate chemical reactions between the starch decomposition products and the poly(cinnamyl methacrylate) covalently bonded to its structure.

Above the temperature of 340 °C, one main, endothermic DSC peak for the tested copolymers is observed. Its intensity is directly dependent on the G value. As the G value increases, its intensity and therefore the area under this peak also increase. This suggests that it is related to the decomposition processes of poly(cinnamyl methacrylate) chains. This supposition by the course of the DSC curve for the poly(cinnamyl methacrylate) homopolymer is confirmed. Above the temperature of 340 °C, an endothermic DSC signal with a maximum at 420 °C is also observed, Figure 3.

### 2.5. The Thermal Stability and Thermal Decomposition Course in Inert Atmosphere

**Figure 4:**
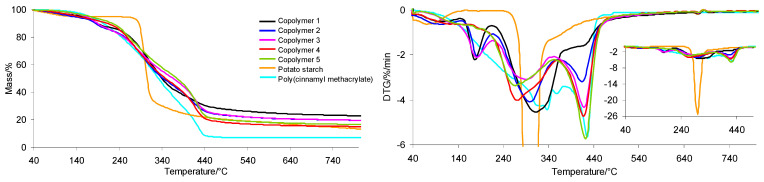
The TG/DTG curves for the starch-g-poly(cinnamyl methacrylate) copolymers, potato starch and poly(cinnamyl methacrylate) in inert atmosphere.

**Figure 5:**
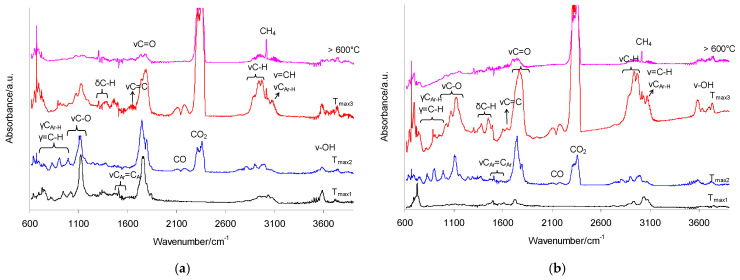
The gaseous FTIR spectra for the copolymers 1 (**a**) and copolymer 5 (**b**) ininert atmosphere.

The thermal curves obtained under the heating of the copolymers, potato starch and homopolymer in the presence of an inert atmosphere are presented in Figure 4. Generally, all the copolymers are thermally stable up to the temperature of 150 °C. Below this temperature, only the mass loss (Δm_0_) connected with the moisture evaporation is indicated. The heating of the novel materials above their thermal stability results in their decomposition process. According to the TG/DTG curves, these materials decompose at three main temperature ranges. The first one between the temperatures 150 °C and 220 °C with a maximum temperature at 177–185 °C is visible. The mass loss (Δm_1_) varies between 2.3% to 12.6%, shown in Table 4. According to the gaseous FTIR spectrum collected at this temperature range, the creation of some organic fragments is affirmed based on the presence of the following absorption bands: at 3581 cm^−1^ (the stretching vibrations of the OH), at 3016–3031 cm^−1^ (the stretching vibrations of the C_Ar_-H), at 3054–3056 cm^−1^ (the stretching of the =C-H), at 2877–2956 cm^−1^ (the stretching vibrations of the C-H), at 2703–2800 cm^−1^ (the stretching vibrations of the C-H in aldehyde group), at 1718–1820 cm^−1^ (the stretching vibrations of the C=O), at 1508–1598 cm^−1^ (the stretching vibrations of the aromatic C=C), at 1336–1382 cm^−1^ (the deformation vibrations of the C-H), at 1010–1240 cm^−1^ (the stretching vibrations of the C-O) and at 634–937 cm^−1^ (the out of plane deformation vibrations of the =C-H and the C_Ar_-H), shown in Figure 5. The obtained FTIR data suggest the creation of some aldehyde, acid, ester, alcohol, aromatic and alkene fragments as the result of depolymerization processes of poly(cinnamyl methacrylate) chains with lower polymerization degree. In the case of the unmodified potato starch decomposition, no mass loss in this temperature range is observed. However, slow mass loss without a clearly marked maximum for poly(cinnamyl methacrylate) decomposition, in the mentioned temperature range, is observed. Therefore, it can be assumed that the mass loss (Δm_1_) in this temperature range to the decomposition of poly(cinnamyl methacrylate) chains with lower polymerization degree attached to starch backbone is related. At this temperature range, unmodified potato starch is thermally stable. Of course, due to the complicated structure of the obtained copolymers and multi-path decomposition reactions, it can be said that depolymerization is the main reaction. In addition, other reactions may take place, such as an accidental breaking of the bonds, reactions between decomposition products in the gas phase or reactions between gaseous products and the resulting residues.

The second decomposition stage from the temperature ca. 220 °C to ca. 360–370 °C with the maximum temperature at 265–312 °C appeared. This decomposition stage by one broad, asymmetrical DTG signal is described. This suggests some of the simultaneous decomposition reactions including the cleavage of the bonds in the structure of the graft copolymers. The mass loss (Δm_2_) is from 32.1% to 48.1%. At this decomposition stage, mainly the creation of the volatiles originating from the decomposition of starch based on the gaseous FTIR spectrum is observed. The absorption bands are characteristic for the stretching vibrations of the OH groups above 3500 cm^−1^ with two main maxima at 3573 cm^−1^ and at 3721 cm^−1^, the bands for the stretching vibrations of the =C-H at 3020–3050 cm^−1^, the bands for the stretching vibrations of the C-H at the range of 2794–2973 cm^−1^, the bands for the stretching vibrations of the C-H in aldehyde groups at 2715 cm^−1^, the bands at 1743–1768 cm^−1^ responsible for the stretching vibrations of the C-O, the bands at the range of 1500–1546 cm^−1^ from the stretching vibrations of the aromatic C=C, the bands at 1344 cm^−1^ and 1445 cm^−1^ from the deformation vibrations of the C-H, the bands at the range of 1024–1290 cm^−1^ (the stretching vibration of the C-O) and the bands below 990 cm^−1^ (the out of plane deformation vibrations of the =C-H). The presence of these absorption bands points to the emission of acid, aldehyde, alkane, alkene, alcohol and aromatic fragments under the pyrolysis process of starch. Furthermore, the creation of water (the bands above 3500 cm^−1^), CO_2_ (the bands at 2300–2360 cm^−1^ and at 669 cm^−1^) and CO (the bands at 2080 cm^−1^ and 2160 cm^−1^) confirms the dehydration and decarboxylation of starch and their intermediate decomposition products. In a similar temperature range with a maximum temperature at 299 °C with a tail arm at 372 °C, the decomposition of unmodified potato starch is observed. Therefore, the results obtained from the gaseous FTIR analysis and the results from the TG/DTG analysis of unmodified potato starch confirm that in the above temperature range, the starch from the copolymers decomposes. However, due to the existence of broad, asymmetric DTG signal, the formation of some products as a result of depolymerization and degradation of poly(cinnamyl methacrylate) chains with higher polymerization degree cannot be excluded. The appearance of the bands responsible for the characteristic vibration for the decomposition products of poly(cinnamyl methacrylate) may overlap with those characteristic for the starch decomposition products. As is visible, the decomposition of unmodified potato starch and the decomposition of the homopolymer run in a similar temperature range, shown in Figure 4. The maximum DTG decomposition temperature of poly(cinnamyl methacrylate) with three maxima at 317, 340 °C and 363 °C is visible. The mass loss for the homopolymer is significant (67.3%). However, due to the shift in the maximum decomposition temperature for the homopolymer towards higher values compared to unmodified potato starch, the highest intensity of gases formed as poly(cinnamyl methacrylate) decomposition products only at temperatures above 340 °C is observed. At a lower temperature, the gaseous FTIR spectrum of potato starch decomposition products is of high intensity, so the volatiles produced as a result of the decomposition of the poly(cinnamyl methacrylate) chains are less visible.

**Scheme 2:**
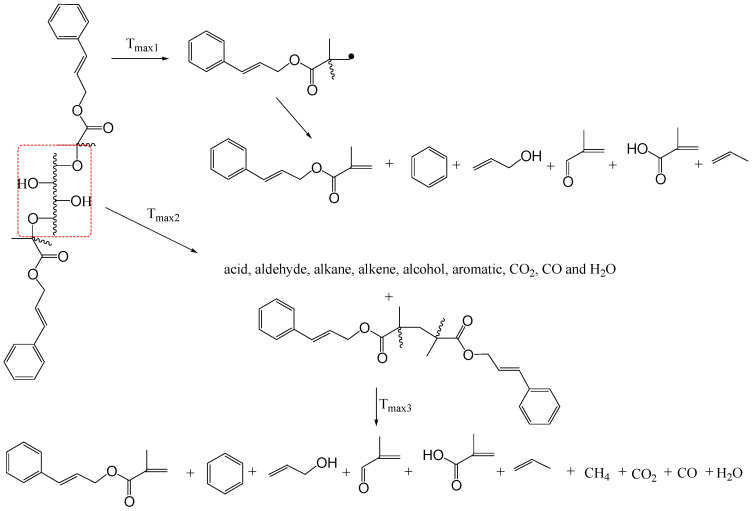
The course of the pyrolysis of the copolymers based on the gaseous FTIR results.

The next decomposition stage from the temperature ca. 360–370 °C to ca. 800 °C with T_max3_ at 417–421 °C for all the studied copolymers appeared. This stage by one, asymmetrical DTG signal with a tail visible at higher temperatures is characterized. The mass loss (Δm_3_) is from 16.7% (copolymer 1) to 38.7% (copolymer 5). It obviously depends on the G values. As the G values increase, the mass loss (Δm_3_) also increases. The gaseous FTIR spectrum collected at this temperature range shows the absorption bands characteristic for the stretching vibrations of the OH above 3500 cm^−1^ with two maxima at 3575 cm^−1^ and 3727 cm^−1^, the stretching vibrations of the =C-H at 3068–3080 cm^−1^ and the C_Ar_-H at 3028 cm^−1^, the stretching vibrations of the C-H at 2877–2968 cm^−1^ and at 2720 cm^−1^ (the C-H at the CHO groups), the stretching vibrations of the C=O at 1764–1781 cm^−1^, the stretching vibrations of the C=C at 1625–1630 cm^−1^, the stretching vibrations of the aromatic C=C at 1493–1511 cm^−1^, the deformation vibrations of the C-H at 1377 cm^−1^ and 1450 cm^−1^, the stretching vibrations of the C-O at 1024–1300 cm^−1^ and the out of plane deformation vibrations of the =C-H and the C_Ar_-H below 900 cm^−1^. Moreover, at this decomposition stage, the bands responsible for the formation of H_2_O above 3500 cm^−1^, CO_2_ at 2314–2358 cm^−1^, CO at 2078–2160 cm^−1^ and CH_4_ at 3014 cm^−1^ are observed [42,43,44,45]. The results show that during this decomposition stage the depolymerization processes of the grafted poly(cinnamyl methacrylate) with high polymerization degree connected with the further decomposition processes such as decarboxylation, and condensation of the intermediate gaseous species, as well as subsequent pyrolysis, dehydration, decarboxylation of the previously formed residues are expected. This is in agreement with the TG/DTG curves obtained for poly(cinnamyl methacrylate). The further decomposition of this homopolymer in this temperature range with the maximum temperature at 427 °C and a mass loss of approx. 25.5% is observed. As a consequence, the creation of various structure volatiles, among them some aromatic, alkane, acid, aldehyde, alcohol, ester fragments, methane, water, carbon dioxide and carbon monoxide, is observed in Scheme 2.

The heating of the starch graft poly(cinnamyl methacrylate) copolymers to the temperature of 800 °C leads to the creation of some of the residue (16.0–22.9%).

### 2.6. The Thermal Stability and Thermal Decomposition Course in Oxidative Atmosphere

**Figure 6:**
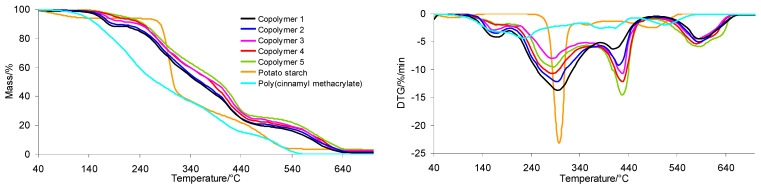
The TG/DTG curves for the starch-g-poly(cinnamyl methacrylate) copolymers, potato starch and poly(cinnamyl methacrylate) in oxidative atmosphere.

**Figure 7:**
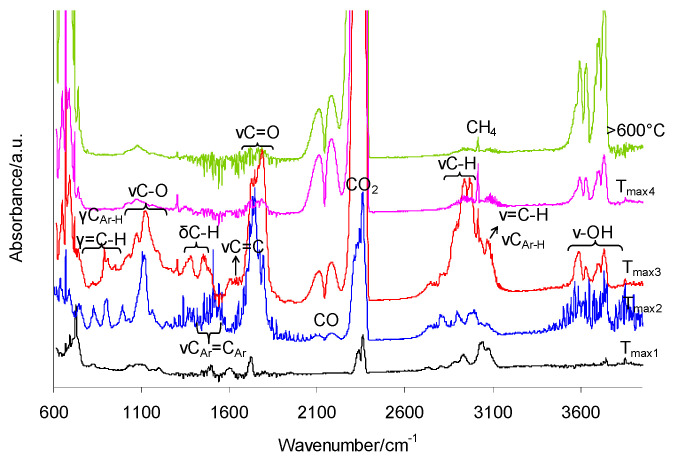
The gaseous FTIR spectra for the copolymers in air conditions.

The TG and DTG curves collected in oxidative conditions for the prepared starch graft copolymers, potato starch and poly(cinnamyl methacrylate) are presented in Figure 6. In addition, the TG/DTG data are placed in Table 5. As is well visible, the tested materials decompose in at least four main stages under the heating in the presence of oxygen. The first decomposition stage by low intensity DTG peak is described. It extends from ca. 90–100 °C to ca. 200 °C with a maximum temperature (T_max1_) at 157–167 °C. The mass loss (Δm_1_) is small (12.3–6.6%) and it decreases with increasing G values. The gaseous FTIR spectra collected at T_max1_ show the presence of the absorption bands responsible for the stretching vibrations of the OH (above 3500 cm^−1^), the C_Ar_-H and the =C-H at 3054–3025 cm^−1^, the C-H at 2707–2920 cm^−1^, the C=O at 1710–1720 cm^−1^, the aromatic C=C at 1536–1580 cm^−1^, the C-O at 1012–1174 cm^−1^, the deformation vibrations of the C-H at 1438–1482 cm^−1^ and the out of plane deformation vibrations of the =C-H and the C_Ar_-H below 900 cm^−1^. The occurrence of these bands indicates the creation of some decomposition products as a result of pyrolysis and oxidation reactions of volatiles formed from low molecular mass poly(cinnamyl methacrylate) chains attached to the starch backbone. These results are consistent with those obtained at T_max1_ for the tests in inert atmosphere. It indicates a similar onset decomposition temperature of the tested copolymers regardless of the used atmosphere. Also, in this temperature region, the first decomposition stage of poly(cinnamyl methacrylate) is observed. It confirms that this stage is directly connected with the presence of poly(cinnamyl methacrylate) chains. The next decomposition stage spreads from ca. 200 °C to ca. 360 °C (copolymers 1, 2 and 3) and to 380 °C (copolymers 4 and 5) with T_max2_ at 281–292 °C. The mass loss (Δm_2_) directly depends on the G values. It is from 44.1% (copolymer 1) to 31.7% (copolymer 5). Based on the gaseous FTIR spectra collected under the second decomposition stage, the presence of the absorption bands characteristic for the stretching vibrations of the OH (above 3500 cm^−1^), the =C-H and the C_Ar-_H at 3021–3066 cm^−1^, the C-H at 2728–2965 cm^−1^, the C-O at 1724–1783 cm^−1^, the aromatic C=C at 1520–1546 cm^−1^, the C-O at 1012–1234 cm^−1^, the deformation vibrations of the C-H at 1338–1480 cm^−1^ and the out-of-plane deformation vibrations of the =C-H and the C_Ar-_H at 636–991 cm^−1^ is confirmed, Figure 7. This proves the formation of volatiles, mainly as a result of starch decomposition process. Among these volatiles, the most expected are acid, aldehyde, alkane, alkene, alcohol, aromatics and its oxidized forms. Also, the creation of H_2_O, CO_2_ and CO confirms the dehydration and decarboxylation processes of starch and the formed volatiles under the heating. However, this second decomposition stage spreads over high temperature region and partially overlaps with the decomposition of the homopolymer. Due to this, it can be concluded that, apart from the decomposition of starch, in this temperature range the breaking of the bonds present in the structure of poly(cinnamyl methacrylate) chains and reactions between formed volatiles and oxygen also take place.

**Scheme 3:**
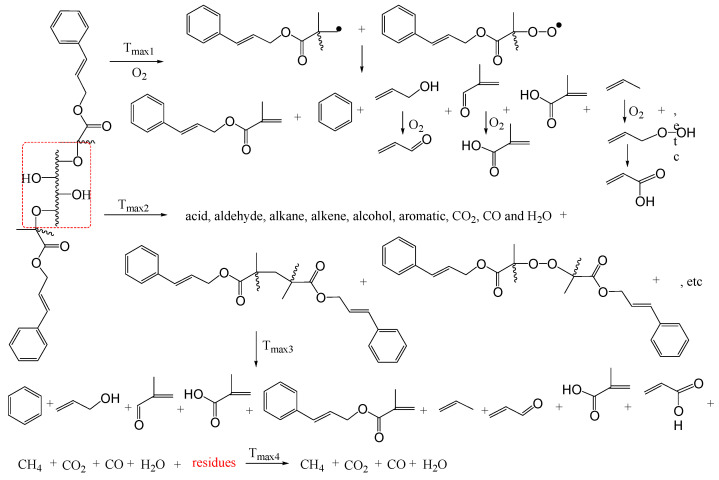
The course of the oxidative decomposition of copolymers based on the gaseous FTIR results.

The third decomposition stage between 360 and 380 °C and 490 °C with T_max3_ at 415–429 °C and the mass loss (Δm_3_) from 24.4% to 36.6% is visible. The Δm_3_ increases as G values increase. These results indicate the decomposition process of poly(cinnamyl methacrylate) chains in this temperature range. As is well visible, the DTG peak for starch graft copolymers and the DTG peak for the homopolymer almost at the same maximum temperature are observed. The FTIR analysis also confirms the emission of the fragments containing the unsaturated, saturated and aromatic compounds with carbonyl, hydroxyl, carboxyl and/or ester groups. Their creation is based on the appearance of the absorption bands for the stretching vibrations of the OH (above 3500 cm^−1^ with two well-defined maxima at 3581 cm^−1^ and 3727 cm^−1^), the =C-H and the C_Ar-_H at 3021–3066 cm^−1^, the C-H at 2723–2967 cm^−1^, the C-O at 1724–1785 cm^−1^, the C=C at 1600–1640 cm^−1^, the aromatic C=C at 1520–1546 cm^−1^, the stretching of the ring at 1303 cm^−1^, the C-O at 1014–1172 cm^−1^ and the deformation vibrations of the C-H at 1371–1446 cm^−1^, and the out-of-plane deformation vibrations of the =C-H and the C_Ar-_H at 647–944 cm^−1^ is proved. In addition, the creation of H_2_O, CO_2_, CO and CH_4_ (3016 cm^−1^) is confirmed [42,46,47]. It is highly probable that these gaseous decomposition products are the results of the pyrolysis of high molecular mass chains of poly(cinnamyl methacrylate) and the oxidation and combustion of some of the resulting fragments. In this decomposition stage, the combustion processes of the formed residues can also happen. The last decomposition stage extends above temperatures of 490 °C with T_max4_ at 581–587 °C and with the mass loss (Δm_4_) in the range of 18.8–22.0%. In addition, after heating the tested compounds to a temperature of 700 °C, a small residue (1–3.1%) is observed. In this decomposition stage, the formation of inorganic gases mainly occurs, namely, H_2_O, CO_2_ and CO, and some of organics such as CH_4_ (3016 cm^−1^) and compounds with the C-O and the C=O groups are visible. This stage is directly connected with the oxidation and/or combustion processes of the residues formed from both potato starch and poly(cinnamyl methacrylate) chains. As is confirmed, in an atmosphere where oxygen is present, the decomposition process of starch graft copolymers is more complicated compared to their decomposition in inert conditions. This is due to the breaking of the bonds present in the structure of the tested materials, reactions between the gaseous decomposition products and the formed residues with oxygen and the combustion reactions. It leads to the formation of a greater variety of gases than in inert atmosphere, as presented in Scheme 3.

## 3. Materials and Methods

### 3.1. Materials

Cinnamyl methacrylate monomer, in the laboratory of the Department of Polymer Chemistry, UMCS, during the acylation of cinnamyl alcohol (98%, Sigma-Aldrich, Burlington, MA, USA) with methacryloyl chloride (97%, Sigma-Aldrich) in the presence of triethylamine (99%, Sigma-Aldrich) as a catalyst according to the method described in Refs. [38,39], was prepared. Potato starch was first purified from potato flour (Melvit S.A., Olszewo-Borki, Poland) according to the method proposed by Lim et al. [48]. The initiator, potassium persulfate (≥99%), was from Fluka, Buchs, Switzerland. The following solvents, tetrahydrofuran, dichloromethane, chloroform, toluene, hexane, butanol, ethanol and silica gel, were delivered from Merck, Darmstadt, Germany. However, carbon tetrachloride, sodium hydroxide, sodium carbonate, magnesium sulfate, hydrochloric acid and buffers solutions were received from POCh, Gliwice, Poland. 

### 3.2. Methods

A FTIR Tensor 27, Bruker apparatus (Bremen, Germany) worked at the absorption/transmittance mode to collect the FTIR spectra that were exploited. All the FTIR spectra for the copolymers using a KBr disk technique over the region 600–4000 cm^−1^ and 4 cm^−1^ resolution were gathered. A Bruker Avance 300 MSL instrument (Bremen, Germany) was used to gather the ^1^H NMR spectrum of methacrylic monomer. The NMR spectra at the resonance frequency of 300 MHz were collected. The chemical shifts to deuterated chloroform (CDCl_3_), as an internal standard, were referenced. 

Cross-polarization magic angle spinning (CP-MAS ^13^C NMR) measurements with the use of a Brucker Avance 300 MSL instrument (Bremen, Germany) operating at 75 MHz were performed. The spectra using a number of scans 2048 and the spin rate of 8000 Hz were collected. The ability to swell novel starch-g-poly(cinnamyl methacrylate) copolymers in different polarity solvents such as water, ethanol, butanol, toluene, hexane and carbon tetrachloride was studied. The centrifugation method was applied. The swell-ability coefficients (B) of the copolymers based on the equation taken from Ref. [49] were calculated as follows:

B = (V_1_ − V_0_)/V_0_ × 100%,

V_0_ is the initial sample volume, and V_1_ is the final sample volume.

The moisture absorption tests at the temperature of 25 °C with the use of ca. 100 mg of the sample were performed. The sample in a desiccator was placed and exposed to the water vapor. The percent of the moisture absorption (M%) by the copolymers based on the equation was counted [50].

M = m_1_ − m_0_/m_0_ × 100%,

m_0_ is the initial sample mass, and m_1_ is the final sample mass.

The chemical stability of the received copolymers in various chemical environments such as 1M NaOH, 1M HCl and buffer solutions with the following pH 5, 7 and 9 was studied. The tests were carried out until a constant mass of the residues was achieved. The percent of the mass loss (WL) from the equation below was calculated [51] as follows:

WL = (m_1_ − m_2_)/m_1_ × 100%,

m_1_ is the initial sample mass, and m_2_ is the final sample mass.

A DSC 204 Jupiter F1, from Netzsch, Germany, instrument to evaluate the thermal studies of the copolymers, potato starch and homopolymer was used. The analyses in aluminum crucibles equipped with a pierced lid between the temperatures 20 °C and 500 °C and the sample mass ca. 10 mg were conducted. An inert atmosphere (argon, a flow rate 20 mL/min) and the heating rate 10 K/min were applied.

A STA 449 Jupiter F1, Netzsch, Germany, instrument to determine the thermal stability and the decomposition stages of the novel materials, potato starch and homopolymer was employed. The copolymer sample (ca. 10 mg) between 40 °C and 800 °C in Al_2_O_3_ crucible was heated. The heating rate was 10 K/min. An inert atmosphere (helium, a flow rate 40 mL/min) and an oxidizing atmosphere (synthetic air, a flow rate 100 mL/min) were used.

A FTIR TGA 585 analyzer from Bruker, Bremen, Germany, coupled on-line to a STA 449 Jupiter F1 to evaluate the type of the gaseous decomposition products emitted under the heating of the copolymers and thus their decomposition course was used. All the FTIR spectra in absorbance mode and in the range of 600–4000 cm^−1^ with a resolution 4 cm^−1^ were collected.

### 3.3. The Preparation of the Graft Copolymers

The graft copolymers using the following graft conditions, the graft temperature 65 °C, the graft reaction time of 90 min, an initiator concentration of 1.5 wt% and different potato starch to cinnamyl methacrylate ratio, were synthesized, shown in Table 1. The applied graft conditions were an optimal based on the preliminary tests where various graft temperature, graft reaction time and an initiator concentration were applied. The graft copolymerization of potato starch with cinnamyl methacrylate in the glass flask equipped with a thermometer, a glass stirrer and a balloon with nitrogen was carried out. Initially, potato starch and distilled water (50 mL) were heated to 65 °C for 30 min to starch gelation. After 30 min, 1.5 wt% of the water soluble initiator was added and heated for the next 30 min under inert atmosphere. Finally, the methacrylate monomer was dropped and the reaction mixture for was heated 90 min. After the specified graft reaction time, the whole reaction mixture was transferred to methanol (150 mL) to precipitate. The raw product was drained off under the reduced pressure, washed several times with methanol and distilled water and dried at 60 °C. After drying, the raw product was purified by a Soxlet extraction method with tetrahydrofuran as the solvent in order to remove the formed homopolymer.

## 4. Conclusions

The performed studies allowed for the preparation of highly resistant starch graft copolymers using the “grafting from” method. As the amount of cinnamyl methacrylate monomer in the mixture with starch increased, the G values increased. The copolymers with the highest G value using a starch to monomer ratio of 1:3 under the used experimental conditions were obtained. Based on performed tests, we found the following:

- The swelling of the copolymers changed with changing G values. As the G value increased, the swelling of the copolymers decreased in the polar solvents and increased in the non-polar solvents. Their swelling in the polar solvents was definitely lower compared to the swelling of unmodified potato starch, while the swelling of the copolymers in non-polar solvents was higher compared to the swelling of starch.
- The moisture absorption of the copolymers changed with changing G values. As the G value increased, the moisture absorption of the copolymers decreased. The moisture absorption of the copolymers was definitely lower than the moisture absorption of unmodified starch.
- The chemical stability of the copolymers changed with changing G values. As the G value increased, the chemical stability of the copolymers in acidic, alkaline and neutral environments increased. The chemical stability of all the tested starch graft poly(cinnamyl methacrylate) copolymers was definitely higher, especially in an acidic and neutral environments as compared to the chemical stability of unmodified starch.
- The DSC and TG/DTG tests proved that the pyrolysis process of the prepared copolymers took place in at least three main stages. The first stage connected with the pyrolysis process of low molecular mass poly(cinnamyl methacrylate) chains up to temperatures of approx. 220 °C was visible. The second stage described the decomposition of starch from the copolymers happened between 220 °C and 360 °C. The third one with the pyrolysis of high molecular mass poly(cinnamyl methacrylate) chains was connected and it was spread from 360 °C to 600 °C.
- The TG/DTG studies confirmed that the oxidative decomposition of the tested copolymers took place in at least four stages. The first up to 190 °C was due to the oxidative decomposition of low molecular mass poly(cinnamyl methacrylate) chains. The second stage extended between 190 °C and 360–380 °C was a result of the decomposition of starch. The third stage from 360 to 380 °C to 500 °C with the oxidative decomposition of high molecular mass poly(cinnamyl methacrylate) chains was connected. The last one above 500 °C was due to the combustion process of the residues.
- The simultaneous TG/FTIR showed the formation of aldehyde, acid, ester, alcohol, aromatic, alkene, alkane, H_2_O, CO_2_, CO and CH_4_ under the heating of the copolymers in inert conditions. In oxidative conditions, due to the reactions with oxygen, more diverse gaseous products were created. Among them, unsaturated and saturated compounds with carbonyl, hydroxyl, carboxyl and/or ester groups, alkane, alkene and aromatics and its oxidized forms, H_2_O, CO_2_ CO and CH_4_, were emitted. These results indicated the radical decomposition mechanism of the tested copolymers in both atmospheres.
- Due to their properties, the prepared starch graft poly(cinnamyl methacrylate) copolymers can be valuable materials for practical applications, in particular, for pharmaceutical applications as drug coatings or in drug delivery systems and/or as a surface sizing agent or for coating the surface of paper to protect it against external factors.

## Figures and Tables

**Table 1:** The values of grafting percent (G, %).

Copolymer No.	Starch to Monomer Ratio	G/% ^1^
1	1:1	15.3 ± 0.3
2	1:1.5	30.1 ± 0.4
3	1:2	40.2 ± 0.5
4	1:2.5	46.7 ± 0.7
5	1:3	55.3 ± 0.4

^1^ %G = m_3_/m_1_ × 100%, where %G—the grafting percent, m_1_—the mass of potato starch and m_3_—the mass of the grafted poly(cinnamyl methacrylate).

**Table 2:** The copolymer swelling (B, %) and the moisture absorption (M, %).

Copolymer No.	Water	Ethanol	Butanol	Toluene	CCl_4_	Hexane	M (%)
1	20 ± 2	15 ± 2	12 ± 2	12 ± 1	15 ± 2	15 ± 1	18 ± 0.3
2	15 ± 2	11 ± 2	8 ± 2	15 ± 1	17 ± 1	18 ± 1	10 ± 0.4
3	11 ± 1	10 ± 1	7 ± 2	20 ± 2	20 ± 1	20 ± 2	8 ± 0.1
4	11 ± 1	8 ± 1	5 ± 1	20 ± 2	22 ± 2	25 ± 2	5 ± 0.2
5	10 ± 1	8 ± 1	5 ± 1	22 ± 1	25 ± 2	25 ± 1	5 ± 0.1
Potato starch	70 ± 3	55 ± 4	39 ± 2	5 ± 1	8 ± 1	9 ± 1	50.3 ± 0.8

**Table 3:** The chemical stability (WL, %) of the tested copolymers.

Copolymer No.	WL (%)				
	1M HCl	pH 5	pH 7	pH 9	1M NaOH
1	20.2 ± 0.4	14.6 ± 0.5	12.6 ± 0.3	62.4 ± 0.6	63.2 ± 0.5
2	11.3 ± 0.3	9.8 ± 0.4	9.2 ± 0.1	59.3 ± 0.5	60.8 ± 0.5
3	10.1 ± 0.3	9.2 ± 0.2	8.8 ± 0.2	51.6 ± 0.4	59.7 ± 0.4
4	9.3 ± 0.2	8.1 ± 0.2	8.0 ± 0.1	50.1 ± 0.4	58.5 ± 0.3
5	7.4 ± 0.1	5.3 ± 0.2	4.4 ± 0.2	43.2 ± 0.5	55.7 ± 0.3
Potato starch	81.4 ± 0.5	65.5 ± 0.3	62.7 ± 0.5	86.7 ± 0.4	100 (g)

Where g—gelation.

**Table 4:** The TG/DTG data in inert here.

Copolymer No.	T_max0_ /°C	Δm_0_ /%	T_max1_ /°C	Δm_1_ /%	T_max2_ /°C	Δm_2_ /%	T_max3_ /°C	Δm_3_ /%	rm
1	101	4.6	177	8.4	312	48.1	418	16.7	22.9
2	101	5.7	183	8.8	299	39.7	417	26.7	19.1
3	100	2.5	183	12.6	296	32.1	417	33.7	19.1
4	120	6.4	185	3.2	271	39.4	418	35.0	16.0
5	132	4.9	185	2.3	265	37.5	421	38.7	16.6
Potato starch	75	4.4	-	-	299/372	82.2	-	-	13.4
Poly(cinnamyl methacrylate)	-	-	-	-	317/340/ 363	67.3	427	25.5	7.2

**Table 5:** The TG/DTG data in oxidative atmosphere.

Copolymer No.	T_max1_ /°C	Δm_1_ /%	T_max2_ /°C	Δm_2_ /%	T_max3_ /°C	Δm_3_ /%	T_max4_ /°C	Δm_4_ /%	rm
1	165	12.3	292	44.1	415	24.4	586	20.6	1.4
2	164	11.1	289	43.7	420	25.4	587	18.8	1.0
3	160	10.6	283	34.1	428	30.9	581	21.9	2.5
4	157	6.9	281	35.7	427	35.3	581	20.0	2.1
5	167	6.6	289	31.7	429	36.6	585	22.0	3.1
Potato starch	76.7	4.8	297/356	67.8	-	-	492	23.9	3.5
Poly(cinnamyl methacrylate)	166	-	228	63.6	386/416	21.9	521	14.8	0.0

## Data Availability

Data is contained within the article.
